# Estimates of Forest Biomass Carbon Storage in Liaoning Province of Northeast China: A Review and Assessment

**DOI:** 10.1371/journal.pone.0089572

**Published:** 2014-02-25

**Authors:** Dapao Yu, Xiaoyu Wang, You Yin, Jinyu Zhan, Bernard J. Lewis, Jie Tian, Ye Bao, Wangming Zhou, Li Zhou, Limin Dai

**Affiliations:** 1 State Key Laboratory of Forest and Soil Ecology, Institute of Applied Ecology, Chinese Academy of Sciences, Shenyang, China; 2 College of Forestry, Shenyang Agricultural University, Shenyang, China; 3 Institute of Forestry Investigation and Planning, Shenyang, China; 4 Agricultural Technology Promotion Center of Qianshan District, Anshan, China; Chinese Academy of Sciences, China

## Abstract

Accurate estimates of forest carbon storage and changes in storage capacity are critical for scientific assessment of the effects of forest management on the role of forests as carbon sinks. Up to now, several studies reported forest biomass carbon (FBC) in Liaoning Province based on data from China's Continuous Forest Inventory, however, their accuracy were still not known. This study compared estimates of FBC in Liaoning Province derived from different methods. We found substantial variation in estimates of FBC storage for young and middle-age forests. For provincial forests with high proportions in these age classes, the continuous biomass expansion factor method (CBM) by forest type with age class is more accurate and therefore more appropriate for estimating forest biomass. Based on the above approach designed for this study, forests in Liaoning Province were found to be a carbon sink, with carbon stocks increasing from 63.0 TgC in 1980 to 120.9 TgC in 2010, reflecting an annual increase of 1.9 TgC. The average carbon density of forest biomass in the province has increased from 26.2 Mg ha^−1^ in 1980 to 31.0 Mg ha^−1^ in 2010. While the largest FBC occurred in middle-age forests, the average carbon density decreased in this age class during these three decades. The increase in forest carbon density resulted primarily from the increased area and carbon storage of mature forests. The relatively long age interval in each age class for slow-growing forest types increased the uncertainty of FBC estimates by CBM-forest type with age class, and further studies should devote more attention to the time span of age classes in establishing biomass expansion factors for use in CBM calculations.

## Introduction

From a global perspective forest ecosystems account for 80% of biomass carbon of terrestrial vegetation and play an important role in carbon cycling in terrestrial ecosystems [1]. Forest biomass carbon is significantly affected by timber harvesting, land use, climate change and other natural and human-induced disturbances [2]. Given that forests are the major carbon sink in China, the accurate estimate of forest carbon storage and its change is critical for understanding the budget with respect to China's CO_2_ emissions, as well as for scientific assessment of the effects of forest management on the capacity of forests to act as carbon sinks.

China's continuous forest inventories began in 1973 and now have completed seven rounds, each covering a five-year period. Detailed information is gathered on forest area and merchantable timber volume (stems with DBH ≥5 cm) by forest type and age class. Since the early 1980s, the data have been widely used to calculate forest biomass carbon (FBC) storage at regional and national scales [3]–[7].

There are three kinds of methods that are generally used for calculating estimates of FBC. The mean biomass density method [MBM] directly measures biomass in sample plots and uses the average of total plot biomass for each forest type (or forest type with age class) to get biomass per hectare for that type, requiring only inventory data on forest area. As described by Guo et al [8], the mean ratio method [MRM] uses the ratio of plot biomass/ha to plot volume/ha to calculate FBC, requiring only inventory data on forest volume. This requires calculation of a biomass expansion factor (BEF) that converts stem volume to biomass to account for all tree components (i.e, branches, roots, and leaves in addition to stem). BEF is the ratio of all stand (or plot) biomass to stem volume, and the mean ratio method [MRM] for estimating FBC utilizes a mean BEF value in so doing.

The continuous biomass expansion factor method [CBM] expands on the above method by treating BEF not as a constant for a stand in BEF calculation, but by recognizing that BEF actually varies with forest age, stand density, and type of site. To address this variation, Fang et al. [4], [9], [10], [11] derived an equation that accommodates changing BEF values over time from inventory data on forest area and volume. This has been called the continuous BEF method, or more commonly, CBM.

A previous study found that CBM underestimates FBC, MBM overestimates FBC, while MRM generates values similar to, but slightly larger than those from CBM [8]. Forest age significantly influences estimates derived from MBM and MRM: the former overestimates FBC for young forests and underestimates it for old forests, while the reverse is true for MRM [8]. The various estimates derived from the methods for the same forest types provided additional support that the differences in estimates from these methods is actually related to age structure [8], [12]. Thus for a given forest inventory data set, estimates of FBC utilizing the three calculation methods vary greatly, with the maximum estimates almost twice as large as minimum estimates. These results from different methods serve to cloud the exact status of FBC, especially when there exists large areas of young or mature forests.

MBM may overestimate FBC because investigators subjectively prefer selecting healthy (i.e., well-growing) sample forest plots. Approaches to estimating BEF (used in MRM and CBM methods) currently being employed in China may be classified into 3 types based on scope and/or substantive focus – BEF as a regional average (not considering forest types and age classes); BEF by forest type; and BEF by forest type with age classes. Clearly a smaller scale enhances the likelihood of more accurate BEF estimates. However, the heavy workload required for field investigation often precludes research at such smaller scales. To date most of the estimation approaches have been applied the national or regional level.

Applying the approaches at the provincial level may result in large differences in FBC estimates, due primarily to two factors. The first pertains to the grouping of forest types. Most of the studies have found that BEF varies with forest type, site and tree age [3], [4], [8]. However, the BEF in these studies on FBC at the national scale divide all tree species into at most 36 forest groups [13], despite the fact that there are about 2000 tree species in China, with over 100 major forest types, and dominant forest types differ in different provinces. Diverse groupings of forest types will thus generate a variety of estimated carbon values.

The second major factor related to FBC estimates at the provincial level concerns age classes. As mentioned above, the estimates yielded by the three methods may differ greatly for particular age classes, and tree age and size are the major factors [14]. If young and middle-age forests (or mature and over-mature forests) constitute a high proportion of both area and volume in some provinces, the divergence among carbon estimates will further increase.

To date researchers have estimated the FBC storage in Liaoning Province using the approaches established at a regional scale, and the maximum estimated value is almost three times that of the minimum[15], [16], [17]. There have as yet been no studies on FBC storage in Liaoning Province utilizing BEF methods (i.e., MRM or CBM) based on sample plot data derived entirely from sites within the province. Thus it is difficult to assess the accuracy of values estimated from different approaches.

Given the predominance of young and middle-age forests in the province, this paper hypothesized that current estimates of FBC in Liaoning Province are not accurate. To test it and better understand FBC storage in Liaoning Province, we: 1) compare strengths and shortcomings among different methods for estimating FBC; 2) compare FBC estimates and their variation by age class from published studies to those derived from approaches designed in this study utilizing data derived entirely from sample plots within the province and incorporating the grouping of forest types according to those actually found in the province; and c) briefly address future research needs.

## Data and Methods

### 1 Study area

Liaoning Province, located in the southern part of Northeast China (38°43′–43°26′ N, 118°53′–125°46′ E), experiences a monsoon climate of the temperate zone. Annual mean temperature is 5.2–11.7°C and annual total precipitation is 400–970 mm.

According to China's Seventh National Forest Inventory (2004–2008), the forest area in Liaoning Province is 3.61 million ha, accounting for 2.3% of the national total. The forest growing stock is 2.02 billion m^3^, which is 1.5% of the national total; and forest cover is 30.8%. Due to long term human disturbance (i.e., harvesting), young and middle-age forests are now extensive in the province, together occupying 77.9% of the forest area and accounting for 56.7% of the forest growing stock [18].

Forests in the eastern part of Liaoning Province account for the largest area and growing stock, some 62.1% and 72.6%, respectively, of the provincial totals. Dominant forest types in the province today consist primarily of: oak forest (*Quercus spp.*), primarily secondary forests occupying 24.6% and 19.8% of the provincial forest area and growing stock, respectively; larch forest (*Larix spp.*), mostly plantations, accounting for 9.5% and 13.6% of provincial forest area and growing stock, respectively; Chinese pine forest (*Pinus tabulaeformis*), approximately 13.6% and 10.2% of provincial forest area and growing stock, respectively; poplar forest (*Popula spp.*), mostly plantations, accounting for 9.9% and 10.7% of forest area and growing stock, respectively, in the province; and broadleaved mixed forest, mostly secondary forest, occupying 21.5% and 36.9% of provincial forest area and growing stock, respectively[18]. In 2010 these five forest types together occupied 79.1% of the forest area and 84.1% of the forest growing stock in Liaoning Province.

### 2 Data collection

#### 2.1 Forest inventory data

China's Continuous Forest Inventory periodically documents information on the country's forest area and timber resources. The data used in this study includes that collected in six national forest inventories (the 2^nd^ to 7^th^ National Inventories) 1977–1981, 1984–1988, 1989–1993, 1994–1998, 1999–2003 and 2004–2008 [18]–[24]; as well as data on Liaoning Province from the 8^th^ National Forest Inventory (2009–2013) [25]. Inventory data includes area and growing stock by age class for each forest type. After 1980, five age groups are distinguished in the National Inventory: young forest, middle-age forest, near-mature forest, mature forest and over-mature forest. Prior to 1980 only three age groups had been identified: young forest, middle-age forest, and mature forest. In this study, these data for Liaoning Province were selected for examining estimates of FBC storage.

#### 2.2 Field investigation data

This work was conducted based on Forestry Standards “Observation Methodology for Long-term Forest Ecosystem Research” of People's Republic of China(LY/T 1952–2011). All our field work in each location has been conducted in the public forest land. For any locations/activities for which specific permission was not required. All our field work has just focused on the vegetation investigation; therefore, the field work in this study did not involve endangered or protected species and provide the specific location of our study.

In order to calculate biomass expansion factors (BEF) of forest types, sample plots were utilized in Liaoning Province based on the National Inventory data described above. The plots were selected as follows: First, the 11 major forest types in the province, which together account for 98.4% and 98.8% of provincial forest area and growing stock, respectively, were identified (Table 1). Based on similarities to the biological characteristics of dominant tree species, the other minor forest types were combined with the aforementioned major types to form 11 forest groups, as depicted in the overall structure of Table 1.

**Table 1:** Major forest types in Liaoning province and forest type groups in this study.

Forest group	Major forest type			Integrated minor forest types	Number of sample plots *Total* 1 */Field* 2
	Forest type	Area (%)	Stem volume (%)		
1	*Pinus koraiensis*	1.05	1.33	*Picea spp., Abies spp., Cupressus spp.*	46/36
2	*Larix olgensis*	9.53	13.57		89/66
3	*Populus* spp.	9.94	7.68	*Salix spp.*	129/81
4	*Pinus sylvestnis* var. *mongolica*	0.97	0.67		51/39
5	*Robinia pseudoacacia* 3	–	–		35/35
6	*Quecus spp.*	24.57	19.80		109/95
7	*Pinus tabuliformis*	13.56	10.22	*P. densiflora, P. thunbergii*	455/194
8	Other hardwood trees^4^	14.07	8.72		43/41
9	Coniferous and broadleaved mixed forest	2.71	3.12		72/42
10	Coniferous mixed forest	0.53	0.79	Other softwood trees	15/15
11	Broadleaved mixed forest	21.50	32.87		29/25
	Total	98.43	98.77		1073/669

1 : Sum of plots established in this study for field data collection and plots described in published studies

2 : Plots established in this study for field data collection

3 : This species was singled out in the latest forest inventory (FDL, 2013), accounting for 5.44% and 1.54% of provincial forest area and growing stock, respectively.

4 : These include *Fraxinus mandshurica*, *Juglans mandshurica*, *Phellodendron amurense*, *Betula platyphylla*, *Betula costata*, *Ulmus pumila*, *Acer mono* and other hardwood tree species dominant in the forest types listed in national forest inventories.

Each forest group thus includes one of the 11 major forest types in Liaoning Province identified in the National Inventory. Four groups also include one or more minor forest types, included on the basis of similarities to the major type in wood density and growth characteristics. In Liaoning Province, the first four forest groups in Table 1 are almost exclusively plantation forests, while the remaining seven groups are comprised of natural forests. The actual number of age-class types (AC types) within a forest group, which form the basis for estimates of forest biomass density in this study, depends on the number of minor forest types that may have been integrated with the major type to form the group; and whether or not the individual age classes actually exist in Liaoning Province for every forest type that comprises the group (i.e. the major type and any integrated smaller types). This latter criterion can only be assessed empirically (see below).

In illustrating the above, the forest group *Pinus koraiensis* includes this major forest type – which accounts for 1.3% of the total forest area and approximately 1.7% of stem volume in Liaoning Province – plus three minor conifer types included on the basis of similarities in wood density and growth characteristics. The former exists as plantations while the small minor types are found in areas scattered around the province. Only two of the four age classes (which, along with species, form the basis for AC types) are found in the province. On the other hand, the major forest type identified in the National Forest Inventory as broadleaved mixed forest accounts for almost 22% and 33% of the provincial forest area and stem volume, respectively. Three of the four age classes for this forest group are currently found in Liaoning Province.

Based on analysis of National Forest Inventory data, which documents age classes for all forest types, it was determined that 57 AC types are found in the province.

Sample plots as a basis for biomass density calculations were established in each of the 57 AC types. It was pre-determined that at least 10 plots per type were to be established. The actual number of plots established for each AC type depended on the size and location of each type in the province. Thus, for example, a large contiguous area occupied by a single AC type would require a relatively small number of plots (≥10), while a type scattered in many smaller areas would require more plots so as to have at least one plot per area.

The location of plots as part of the above process was accomplished via the use of the Forest Inventory for Design and Planning of Liaoning Province [26]. This covers the entire province by compartments and contains data on forest types by area, location, age class, etc. It is thus more detailed that National Inventory data, although the sum of compartment data for the entire province will yield the same results as the National Inventory. It is also much harder to access, however, than are data from the National Inventory. Thus, for example, all previous studies on biomass density estimation have utilized National Inventory data exclusively.

**Figure 1:**
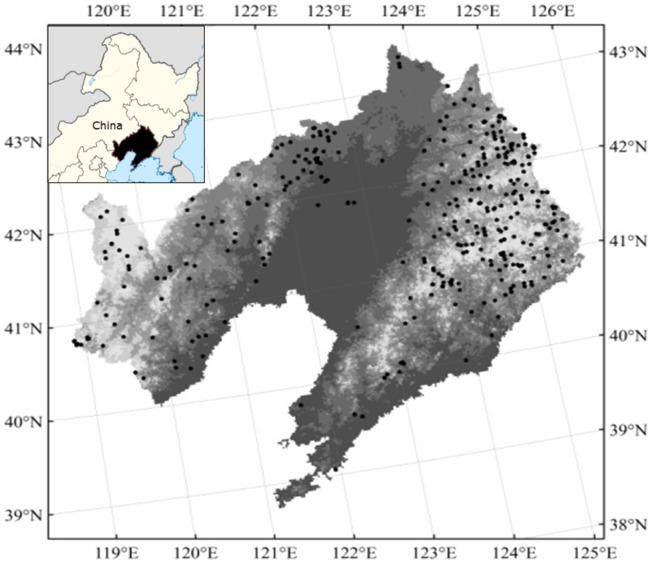
Geographical location, forest flora, and sample sites in Liaoning province.

On an overall basis, sample plots were allocated to 57 forest AC types in Liaoning Province, with at least 10 plots assigned to each AC type, resulting in a total of 669 plots ranging from 0.04–0.1 ha in size (117 plots–20 m×20 m; 498 plots–28.9 m×28.9 m; and 54 plots–20 m×50 m) [Fig. 1, Table 1]. For each plot, diameter at breast height (DBH) and tree height (H) were measured for stems with DBH≥5 cm, following procedures prescribed in China's National Forest Inventory.

This number of sample plots is less than the number that would be expected for this type of study concerned with estimating provincial forest biomass. This in part was one of the major reasons for conducting this study. In order to take advantage of additional data available in published literature, data on 404 sample plots [329 plots–0.06 ha (20 m×30 m) and 75 plots–0.04ha (20 m×20 m)] at 173 study sites in Liaoning Province were collected and merged with that from field investigations. For each of these 404 sample plots, data on stems with DBH recorded as ≥5 cm were retained. The combination of 669 plots established for field data collection and 404 plots whose attributes were described in the literature resulted in a total of 1073 plots from which data was obtained in calculating forest biomass density according to the variety of approaches and methods described below.

### 3 Methods for FBC estimation

#### 3.1 Approaches from published documents

Based on the forest inventory data, the 12 published and widely recognized approaches for FBC estimation in China (Table 2) were chosen to directly calculate the FBC in eight inventory periods in Liaoning Province. In this study, we use the term ‘approaches’ to identify the three major ways in which forest inventory data is used for FBC estimation – regional average, forest type, and forest type with age class. We differentiate these from the three basic ‘methods’ for calculating FBC described earlier – i.e., MBM, MRM and CBM.

**Table 2:** Approaches in published studies for estimating forest biomass carbon.

Authors	Approach	Equations^1^
Wu et al. 2008[32]	Regional MRM	B = V×D/R (D = 0.47, R = 0.567)
Xu et al. 2009[33]	Regional MRM	B = V×δ×ρ (δ = 1.9, ρ = 0.5)
Fang and Chen 2001[34]	Regional -CBM	B = aV+b
Pan et al. 2004 [12]	Regional-CBM	B = aV^b^
Guo et al. 2010[8]	Forest type-MBM	B = BD×A
Guo et al. 2010[8]	Forest type-MRM	B = aV
Zhao and Zhou 2006[31]	Forest type-CBM	B = V/(a+b V)
Fang et al. 2001[4]	Forest type-CBM	B = aV+b
Guo et al. 2010[8]	Forest type-CBM	B = aV+b
Pan et al. 2004[12]	Forest type and age-CBM	B = aV+b
Xu et al. 2007[35]	Forest type and age-CBM	B = aV+b
Xu et al. 2010[13]	Forest type and age-CBM	B = W/(1+k e^−t^)

1 B- forest biomass (Mg ha^−1^); V- forest growing stock (m^3^ ha^−1^); D: stand density; R: the ratio of stem biomass to total biomass; δ: coefficient of stand stem volume to total stand volume; ρ: ratio of biomass to dry weight; A-area (ha); BD- stand biomass density (Mg ha^−1^); t-forest age (years); t: forest age; a,b,k,W- constants.

In published studies, forest types were grouped individually in the process of estimating FBC. In subsequent discussions these forest type groupings are referred to as such, while the term ‘forest group’ is generally reserved for forest type groupings established and utilized in this study.

#### 3.2 Approaches based on field plot data

A total of 43 tree species were recorded in data from the sample plots. Sixteen species-specific allometric equations and stem volume equations based on DBH and/or tree height were available, having been formulated for the major tree species in the region. These formed the basis for 16 tree species groups. The remaining 27 species for which equations were not available were integrated within the 16 species groups following the pattern established in the formation of forest groups in Table 1.

The 16 species-specific allometric equations were then applied to estimate the total forest biomass, including stem, branch, leaf and root [27], [28], [29]. In order to eliminate measurement error, tree height was estimated via a conversion factor from DBH [29]. The stem volume was also estimated utilizing the one-way tree volume tables for Liaoning Province [30]. Biomass expansion factors (BEF) for biomass from forest growing stock were calculated via Eq. (1) according to the continuous biomass expansion factor method (CBM) [Table S1 in file S1] and the mean ratio method (MRM) [Tables S2 in file S1]; and biomass for forest groups and age-class types was then calculated via the CBM method [Eq. (2) and (3), respectively] and the MRM method [Eq. (4) and (5), respectively]. Average biomass density (BD) for forest groups and for age class types was also calculated based on sample plot data (Table S3 in file S1); and then forest biomass was estimated according to the mean biomass density method (MBM) in Eq. (6) and(7).

Biomass expansion factor: 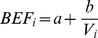 (1)

CBM-forest group: 
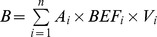
 (2)

CBM-forest group +age class:
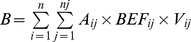
 (3)

MRM-forest group: 
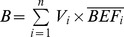
 (4)

MRM-forest group+ age class: 
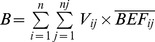
 (5)

MBM-forest group: 
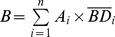
 (6)

MBM-forest group +age class: 
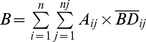
 (7)

where according to forest inventory data

B: total forest biomass in Liaoning Province

n: number of forest groups *nj*: number of age class

and

For forest group *i*: For forest type *i* in age class *j*:

*BEF_i_*: biomass expansion factor *BEF_ij_*: biomass expansion factor

*A_i_*: total area *A_ij_*: total area

*V_i_*: total forest growing stock *V_ij_*: total forest growing stock


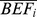
: mean BEF 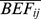: average BEF

*BD_ij_*: biomass density

Carbon storage was estimated utilizing a biomass transformation coefficient of 0.5.

## Results

### 1 Forest resources in Liaoning Province: Four decades of change

**Figure 2:**
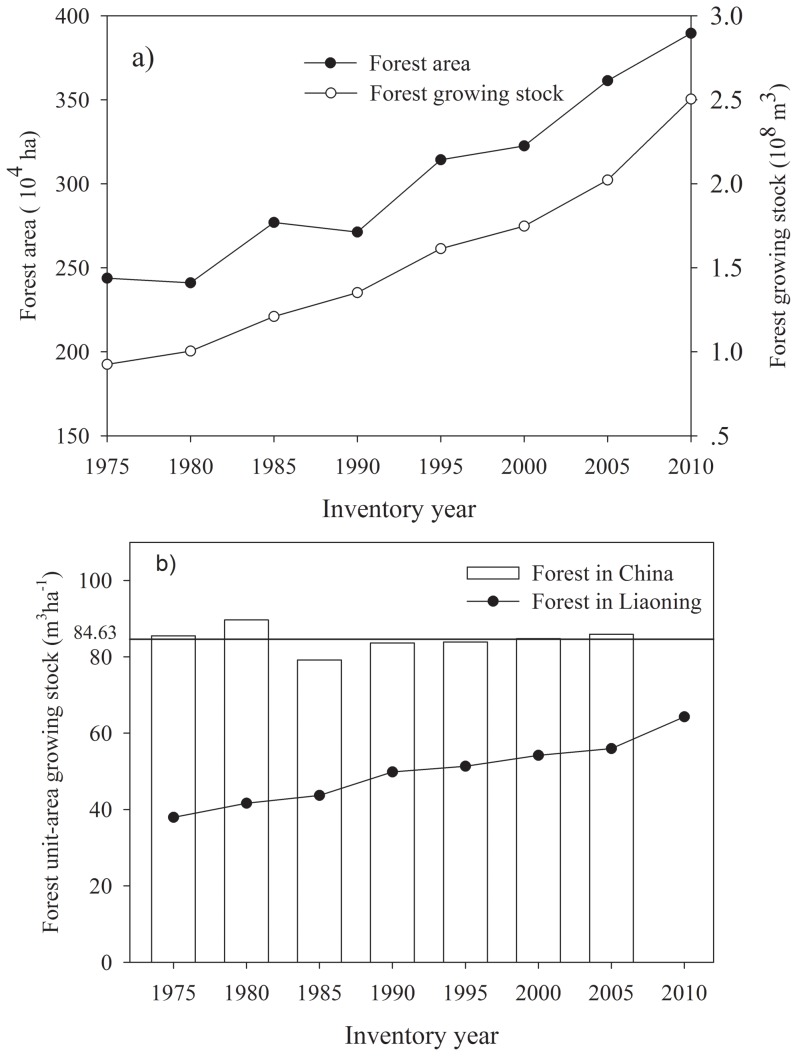
(a) Forest area and growing stock in Liaoning province from 1975–2010; and (b) unit-area growing stock in Liaoning province from 1975–2010. Average unit-area growing stock in China from 1975–2005 was 84.6 m3 ha-1.

Forest cover in Liaoning Province has increased from 18.2% in 1955 to 38.2% in 2010 [18]. Since 1975, forest area and growing stock in the province have continued to increase, from 2.5 million ha and 0.93 billion m^3^ to 3.9 million ha and 2.5 billion m^3^, respectively, in 2010 (Fig. 2a). According to China's Seventh National Forest Inventory, the area and growing stock in 2005 represented 2.3% of the country's forest area and 1.5% of the forest growing stock in China. Although still much lower than the national average, growing stock per hectare in the province increased from 37.9 m^3^ ha^−1^ in 1975 to 56.0 m^3^ ha^−1^ in 2005, and to 64.3 m^3^ ha^−1^ in 2010 (Fig. 2b). Driven by increases in the area and growing stock of mature forests, on an overall basis forest quality has gradually improved.

**Figure 3:**
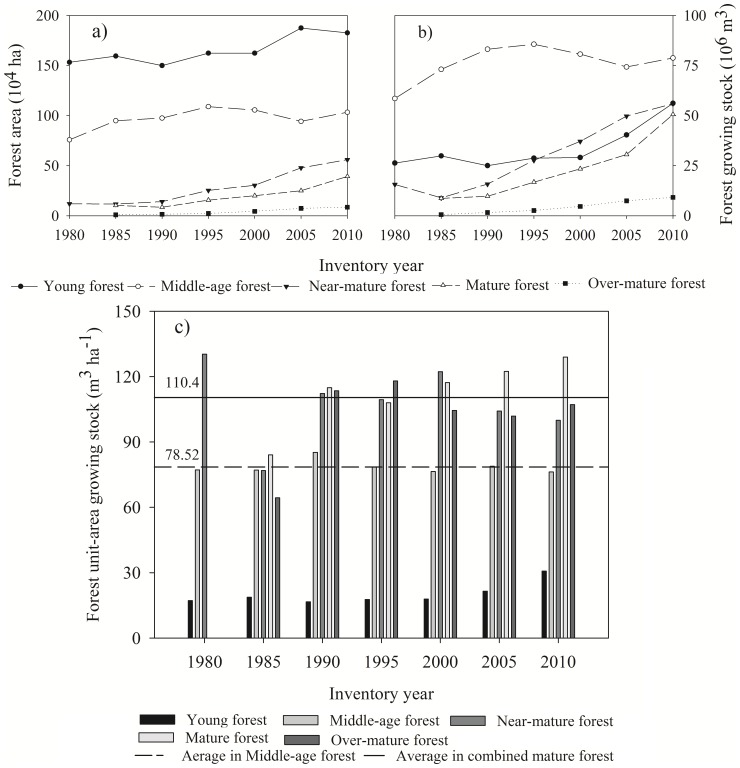
Trajectory of (a) forest area; (b) growing stock; and (c) unit-area growing stock by age class in Liaoning province from 1980 to 2010. Combined mature forest includes near-mature, mature and over-mature forest.

In terms of forest age classes, the area and growing stock of all classes in Liaoning Province increased from 1980 to 2010 (Fig. 3a, b). Near-mature and mature forests displayed much larger increases on a percentage basis than did young and middle-age forests. In 2010, the area of combined mature forests (which includes near-mature, mature and over-mature age classes) was 6.7 times that in 1980, while growing stock was 5.7 times as large. Over this same period, young forests increased by 19.2% in area and 113.4% in growing stock; while forest area and growing stock of middle-age forest increased by 36.3% and 34.6%, respectively. Throughout this period, young forests occupied the largest forest area, followed by middle-age forests, the latter having the highest growing stock during this time. Young and middle-age forests together occupied 95% of total forest area in the province in 1980 and 73.4% in 2010; and 84.5% of total growing stock in 1980, while only 53.8% in 2010. Over-mature forests accounted for the smallest area (2.2% in 2010) and lowest growing stock (3.65%) over this period (Fig. 3b).

Growing stock per hectare of both middle-age forests and combined mature forests (which, as noted above, includes near- and over-mature as well as mature age classes) displayed no distinctive changes from 1980 to 2010 (Fig. 3b), remaining steady at 78.5 m^3^ ha^−1^ and 110.4 m^3^ ha^−1^, respectively. However, since the proportion of combined mature forest increased substantially (from 5% of total forest area in 1980 to 27% in 2010), forest growing stock still increased continuously.

### 2 Forest biomass carbon (FBC) storage by forest group and age class

Results derived from the 12 published approaches to estimating forest biomass carbon (Table 2) showed that the forest type with age class approach generates the highest estimates for FBC, while the regional average approach (forest type and age class not included) yields the lowest estimates (Table 3). Significant differences occur primarily in estimates for young and middle-age forests. There are no significant differences between estimates from forest type approaches and those of forest type with age class (Table 3).

**Table 3:** Average estimate of forest biomass carbon storage (TgC) derived from different approaches based on data from 2^nd^–8^th^ National Forest Inventories.

Approaches	Total	Forest age class				
		Young	Middle	Near-mature	Mature	Over-mature
*Published studies*						
Regional average^1^	81.8(26.7)^C^	21.9(12.6)^B^	35.8(5.1)^B^	15.3(7.0)^A^	12.1(7.2)^A^	1.7(1.4)^A^
Forest type	110.9(42.7)^A^	36.2(30.5)^A^	45.7(6.2)^A^	15.8(8.9)^A^	11.3(10.2)^A^	1.9(1.8)^A^
Forest type with age class	104.4(37.5)^A^	31.5(12.8)^A^	45.1(10.5)^A^	15.16(8.6)^A^	10.9(9.7)^A^	1.8(1.5)^A^
Forest type - MBM*	95.1(29.0)^B^	21.7(10.2)^B^	43.7(4.64)^A^	16.3(8.2)^A^	11.5(9.6)^A^	1.9(1.8)^A^
*In this study*						
Forest group	96.1(21.5)^a^	27.1(4.8)^b^	41.0(2.8)^a^	16.1(8.9)^a^	10.1(8.3)^a^	1.83(1.8)^a^
Forest group with age class	103.5(20.3)^a^	38.6(5.5)^a^	40.7(2.9)^a^	13.9(7.9)^a^	8.7(7.1)^a^	1.61(1.6)^a^
Forest group - MBM *	81.6(19.3)^b^	18.2(5.0)^c^	38.2(3.1)^b^	14.3(8.0)^a^	9.2(7.7)^a^	1.72(1.7)^a^
Forest group with age class - MBM*	82.7(20.3)^b^	18.3(5.2)^c^	38.7(3.0) ^b^	14.6(8.1)^a^	9.5(8.2) ^a^	1.70(1.7)^a^

1 : Based on provincial totals; forest type and age class not used.

* Estimates from mean biomass density method (MBM) methods were eliminated.

Capital letters indicate significant differences in column values for published approaches; small letters indicate significant differences in column values for approaches designed in this study.

With respect to the results from the two approaches designed in this study (forest groups and forest groups with age class), there was also no significant difference in estimates of total FBC between them. However, the estimate for young forests yielded by the forest group approach was significantly less than that of the forest group with age class approach (Table 3).

In general, estimates of FBC from approaches utilizing MBM methods have always generated the highest errors (see discussion below). Therefore, this study also estimated overall average FBC for forest group approaches when MBM estimates were eliminated. Estimates from forest group and forest group with age class approaches that included MBM methods were significantly lower than those from the same approaches with MBM methods eliminated (Table 3). The largest difference occurred between the estimates for young forests. This mirrored results in published approaches with and without the MBM method included.

### 3 Contrasting the three standard methods for estimating FBC

In examining FBC estimates with respect to the three calculation methods from which their parameters are derived – MBM, MRM, and CBM – large differences are apparent (Table 4). MRM generated the lowest estimates of total FBC (average: 81.49 Tg C), while MBM resulted in the highest values (average: 173.82 Tg C), which were 79.2% greater than estimates via CBM and 113.3% greater than those via MRM. Despite large differences in the estimates of total FBC using the three calculation methods, estimates for combined mature forests (i.e., the three higher age classes together) did not differ greatly. The significant differences occurred with respect to young and middle-age forests, especially the former, for which carbon storage estimated by MBM was 3.4 times that by CBM and 5.6 times that by MRM (Table 4).

**Table 4:** Average estimates of forest biomass carbon storage (TgC) utilizing three calculation methods for equation parameters based on data from 2^nd^–8^th^ National Forest Inventories.

Methods	Total	Forest age class				
		Young	Middle	Near-mature	Mature	Over-mature
Published studies						
MBM	173.8(27.5) ^A^	93.9(8.8)A	53.6(4.65) ^A^	13.0(8.9)^A^	11.4(8.6)^A^	1.9(1.8)^A^
MR^c^	81.5(26.7)^C^	16.7(6.0)C	38.1(7.4)^C^	13.4(8.38)^A^	11.4(7.8)^A^	1.9(1.7)^A^
CBM	97.0(32.6)^B^	27.3(12.5)B	42.3(8.0)^B^	13.8(8.64)^A^	11.8(8.7)^A^	1.8(1.6)^A^
MRM-Regional*	98.9 (27.4)^B^	20.3 (6.7)^C^	46.5 (3.6)^B^	13.8 (9.24)^A^	16.1 (10.0)^A^	2.3 (2.1)^A^
CBM-Regional*	99.1 (30.5)^B^	26.8 (7.8)^B^	43.9 (5.7)^B^	12.3 (9.45)^A^	14.3 (9.2)^A^	1.8 (1.7)^A^
In this study**						
MBM	135.1(23.3)^a^	62.0(6.1)^a^	45.7(2.6)^a^	16.19(9.3)^a^	9.6(7.3)^a^	1.7(1.6)^a^
MRM	80.8(20.0)^b^	17.0(5.2)^b^	38.4(3.1)^b^	14.4(8.0)^a^	9.4(8.1)^a^	1.7(1.7)^a^
CBM	83.6(18.9)^b^	19.5(4.8)^b^	38.6(3.0)^b^	14.5(7.8)^a^	9.3(7.5)^a^	1.7(1.7)^a^

MBM: mean biomass density method; MRM: mean ratio method; CBM: continuous biomass expansion factor method.

*: Values derived from regional average approaches were not included. No published studies utilized the MBM method for a regional approach.

**: Average of FBC totals for forest groups and forest groups with age class approaches.

Capital letters indicate significant differences in column values for published approaches; small letters indicate significant differences in column values for approaches designed in this study.

Given that the approaches for FBC estimation (Table 2) that do not include forest type and age class may generate large errors, the estimates from the four regional average approaches (two for CBM and MRM, respectively, since no published studies utilized the MBM method for a regional approach) were removed from the calculation of average total FBC. Re-calculating average total FBC without these regional approaches showed that estimates via the CBM and MRM methods were higher than before and displayed no significant differences between them. At the same time, for young forests significant differences between these two methods were still evident (Table 4).

Recognizing that the different sources of field data utilized in published studies for FBC estimation may generate large differences in FBC estimates, this study used the same field data from Liaoning Province to establish the parameters for CBM, MRM, and MBM; and then utilizing the same set of forest groups based on forest types in the province, estimated total FBC in the province (averaging FBC totals derived from forest groups and forest groups with age class approaches). We found that MBM resulted in the highest estimates, while MRM generated the lowest (Table 4). Significant differences occurred with respect to young and middle-age forests, especially the former. Carbon storage estimated for young forests by MBM was 3.2 times that estimated by CBM and 3.7 times that estimated via MRM (Table 4).

### 4 FBC stocks for Liaoning Province

**Figure 4:**
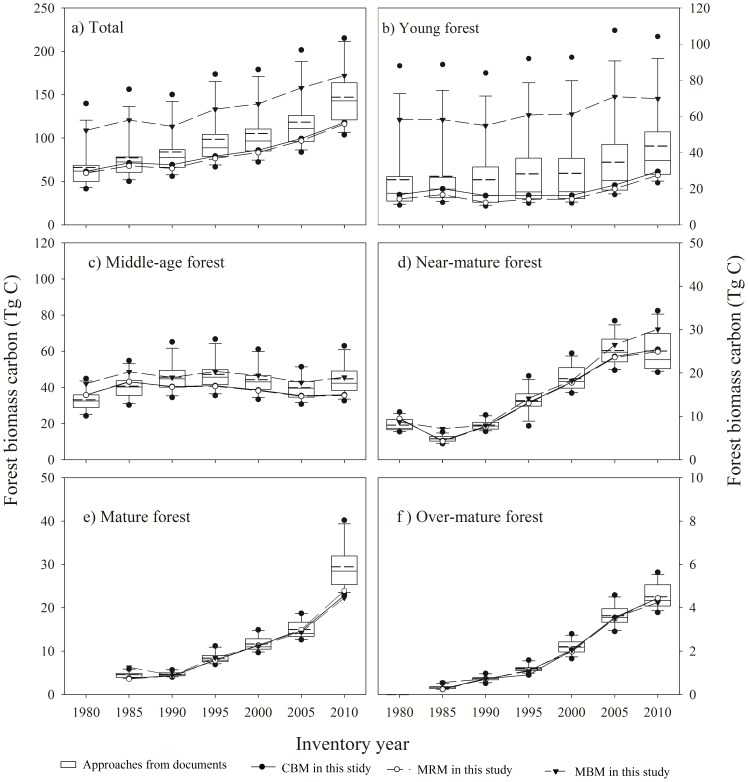
Mean estimates of forest biomass carbon storage in Liaoning province from 1980–2010, derived from 12 published approaches of calculation and approaches established in this study (The values derived from each method represent the average estimate of two approaches by forest groups and by forest groups with age class). The median, 10th, 25th, 75th, and 90th percentiles of estimate from 12 published approaches were plotted as vertical boxes with error bars; the dash lines in the boxes are their average. The top and bottom point (solid circle) are minimum and maximum estimates. MBM: mean biomass density method CBM: continuous biomass expansion factor method; MRM: mean ratio method.

All of the approaches derived from the published documents, as well as those designed in this study, indicate that FBC storage in Liaoning Province has increased over the period of 1980 to 2010 (Fig. 4a). The average estimate of the FBC stocks generated by the 12 published approaches increased from 66.2 (s.d. 25.4) Tg C in 1980 to 147.3 (s.d. 34.1) Tg C in 2010 (Fig. 4a). The magnitude of the FBC estimates varied with different approaches. The character of the standard deviation (sd) accompanying the above estimates reveals that the overall mean estimate divergence derived mainly in connection with young and middle-age forests (Fig. 4b–f). Regarding the FBC in these age classes, the lowest values in each class, which were generated via Wu's regional average MRM method [32], were 14.8% (sd 3.6%); and 58.9% (sd 3.6%) of the highest values in each class, which were found by Guo et al. using the MBM method [8]. The estimates of FBC among the forest group approaches designed in this study (with and without age classes) fell within the range of estimates from published sources (Fig. 4). The difference in estimates derived from published approaches and those designed in this study occurred mainly with respect to young forests. Estimates for other age classes were much closer (Fig.4 c–f).

**Figure 5:**
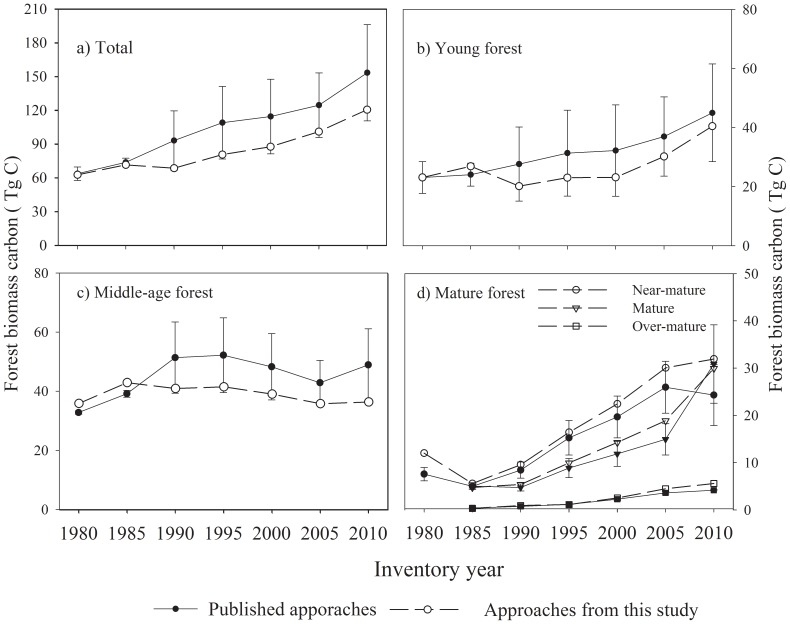
Estimates of forest biomass carbon by CBM (continuous biomass expansion factor method) by forest groups with age class from that in published studies and in this study.

Finally, the CBM methods by forest types with age class from published studies and in the approach established in this study were also used to re-calculate the average FBC. This average in published documents was 20.5% higher than that generated by approaches designed in this study (Fig. 5a). The standard deviations in estimates of published sources were much higher than that of the approach designed in this study. The major differences were again with respect to young and middle-age forests (Fig. 5 b, c, d).

**Figure 6:**
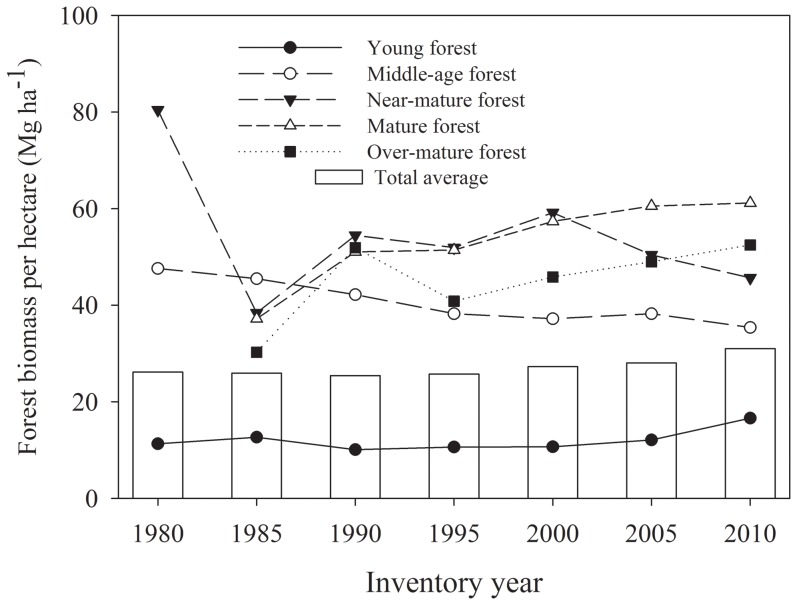
Changes in forest unit-area biomass carbon in Liaoning province in the period of 1980–2010.

Based on the estimates derived from the CBM-forest group with age class approach in this study, total FBC in Liaoning Province has increased over the period of 1980 to 2010 (Fig. 5a). FBC storage increased from 63.03 Tg C in 1980 to 120.87 Tg C in 2010, with an average annual increase of 1.93 Tg C. Examining the trajectories of the FBC storage in different forest age classes over this same period, the annual carbon sink increased by 0.43 Tg C for young forests, 0.53 Tg C for near-mature forests, 0.80 Tg C for mature forests, and 0.15 Tg C for over-mature forests, with a slight increase in middle-age forests of 0.02 Tg C. Thus mature forests displayed the greatest increase in carbon sequestration. In contrast, the FBC storage in young and middle-age forest together decreased from 84.8% of total FBC storage in 1980 to 55.3% in 2010. FBC per hectare increased from 26.15 Mg ha^−1^ in 1980 to 31.02 Mg ha^−1^ in 2010 (Fig. 6), with increases occurring in all age classes except middle-age forests, in which FBC per hectare decreased from 47.59 Mg ha^−1^ in 1980 to 35.38 Mg ha^−1^ in 2010.

## Discussion

This study was motivated by two important needs in Liaoning Province with respect to estimating biomass carbon storage in provincial forests. Each of the published studies on FBC estimation (Table 2) employed one of the three basic methods for estimating FBC – i.e., MBM, MRM, or CBM – and almost all utilized forest types or groups thereof whose parameters, critical for generating FBC values, were determined largely on the basis of data from field plots established outside of Liaoning Province. All data used in the forest group approaches designed in this study utilized data from plots in Liaoning Province to calculate parameters for FBC equations. This greatly enhances the chances that FBC estimates resulting from these approaches better reflect the actual FBC storage in provincial forests.

A second major motivation relates to the utility of these approaches employing forest groups designed in the study. As noted above, field data underlying published studies rely on National Forest Inventory data in calculating FBC. In this study, based entirely on Liaoning field data, FBC was calculated using the three methods noted earlier and compared to results from published studies. The number of plots for each forest group was based on the proportions of provincial forest area and volume by age class occupied by the major species. In addressing this (and the aforementioned) concern regarding the origin of parameters for the FBC equations through the use of forest groups in Liaoning Province, this further strengthens the chances that resultant FBC estimates will better reflect the actual FBC storage in provincial forests.

Without including the estimates generated using MBM methods, it is noteworthy that the lowest values for FBC from published sources (Table 2) were derived from the regional average approaches and higher values from estimates based on forest types with age class. Most of these latter estimates were from studies that included up to 20 forest types for Liaoning Province. The notable exception here was Xu et al. [13], who employed 36 forest types and also treated forest age as a variable (as opposed to employing age classes). It is evident, therefore, that the greater detail employed by Xu et al. [13] contributed the higher estimates of FBC.

It is recognized that in young trees most biomass is allocated to roots, branches and leaves, while for trees in mature growth stages most is allocated to the stem [28]. It is possible, therefore, that approaches by forest types (not differentiated into age classes) may underestimate FBC in young forests. In published studies, the main differences in FBC estimates between approaches using forest types and those using forest types with age class were with respect to young forests (Table 3). However, there were no significant differences among the estimates of FBC in all age classes based on the forest group approaches established in this study (Table 3). This may be related to the large number of field plots for young and middle-age forests in this study, given the large proportion of these age classes in Liaoning Province forests. These two age classes accounted for 39.9% and 28.7%, respectively, of total field plots. Such large proportions may reduce the errors in approaches based on forest groups.

Previous findings have suggested that the continuous biomass expansion factor method (CBM), which takes timber volume as a function of BEF and incorporates effects of forest age, forest density and forest site quality, is more accurate than other methods [8]. In this study, we found a similar result in that, relative to the CBM method, MBM clearly overestimated the value for FBC, especially with respect to young forests (Table 4); while MRM underestimated the biomass carbon for young forests. Thus it is likely that MRM generated the lowest overall value for carbon storage in Liaoning Province due to high proportion of young forests in the province. This result is not consistent with a previous study in which CBM generated the lowest estimates [8]), but is consistent with results described in Fang et al. [3].

Previous studies found that the divergence generated by the different methods can be reduced by the grouping of forest types [13]. Regardless of which calculation method is used to estimate the biomass, prior forest type grouping is necessary, since different model parameters will be associated with different forest type groupings (i.e., forest groups), which in turn may contribute to large gaps in estimated values. To date up to 36 forest types have been employed at the national scale in China, of which only 7–12 can be utilized in classifying forest types in Liaoning Province. Thus even if the same method and same forest inventory data are used, the results obtained from different forest type groupings may be different [4], [15]. It is noteworthy that while forest growing stock in Liaoning Province was larger in 1990 than in 1985 (Fig. 2a), not all estimates of FBC generated by different approaches for 1990 exceeded those for 1985 (Fig. 4a, 5a). The key reason is that in National Inventory data there were only 8 forest types for Liaoning Province before 1985 (the 3rd China's forest inventory), but 18 forest types since 1990 (the 4^th^ China's forest inventory), and 24 forest types in 2010. Consequently, approaches with different forest type groupings yielded large discrepancies in FBC estimates derived therefrom.

This study also found that mean FBC estimates for middle-age forests in published studies displayed different trends than those derived from the forest group approaches developed in this study (Fig. 5c). Further analysis revealed that while estimate trends in published studies were consistent with overall changes in provincial forest growing stock from 1980–2010, the approaches in this study better reflected changes in the growing stock of hardwood forests, resulting in a somewhat lower overall estimate for FBC change over this period (Fig. 5c). In Liaoning Province hardwood forests have a higher proportion of middle-aged forest growing stock and a higher BEF than other forest types. At the same time, from 1985–2010 hardwood growing stock displayed a decreasing trend. At this point, our estimates may be more accurate than those from published documents, since the grouping of forest types employed here better reflects the reality on the ground. Moreover, forest growing stock per hectare in middle-age forests remained almost constant (about 78.5 m^3^) over this period, but the FBC per hectare clearly decreased (Fig. 6). This suggests that our estimates better reflected changes in forest quality in middle-age forests in the province.

Additionally, the species-specific allometric equations for biomass estimates and the one-way tree volume equations for forest growing stock, based on the DBH and/or H from sample plots, were also the important factors affecting the accuracy of estimates. Tree species growing in different climate zones may have different patterns of biomass allocation. This study used data from field plots in Liaoning Province, and the allometric equations and tree volume equations were also established based on data from field plots in the province. Therefore, the estimates derived from the approaches in this study may be more accurate.

With respect to the form of equations used in deriving FBC estimates, most studies employed a liner model for the biomass expansion factor (BEF). Other models were also employed, as exemplified by a power function [12] and hyperbolic functions [31], and these yielded lower FBC estimates than those from linear functions employed by Fang et al. [4] and Guo et al. [8], as well as the linear function used in this study. However, these lower estimates did not differ significantly from those derived from a linear model. This study did not explore the accuracy of the estimates from different ways of equation establishment, but these equation forms undoubtedly affect the accuracy of FBC estimates. Further study in FBC estimation should take these approaches into consideration.

Although estimated values varied depending on the methods employed, the temporal trend of carbon sequestration in forests of Liaoning Province is consistent (Fig. 4a). As discussed above, the forest groups with age class-CBM method utilized in this study may be more accurate than the other approaches and methods which were designed in this study and in published documents. At the same time, our results from the forest groups with age class-CBM approach are quite close to those in some published studies that have employed t the same approach (Fig. 5).

It is worthwhile to note that while the small increase in average carbon storage per hectare of forests in Liaoning Province suggests that forest quality is improving (Fig. 6), this is due primarily to an increase in the proportion of combined mature forests (including near-mature, mature and over-mature forest age classes) (Fig. 3a).The average FBC per hectare in middle-age forests has decreased; in particular, carbon storage in hardwood forests has clearly declined. This almost certainly reflects the fact that middle-age forests constitute the major source of timber production in Liaoning Province.

Mean FBC density in Liaoning Province (27.1 Mg ha^−1^) was still much lower than the national average (about 40 Mg ha^−1^). This is true for all age classes, implying that overall forest quality in Liaoning Province is currently still lower than elsewhere in the country, while also a reminder that that potential exists for improvements in carbon sequestration levels in the province.

As noted above, in order to more accurately estimate forest biomass, we first need to become more proficient at the grouping of forest types. Given that it is unfeasible to determine the biomass expansion factor for every tree species in every age class, further work needs to be done that incorporates major tree species in the region and biological characteristics of individual species. While this study is a beginning, even more explicit criteria for the scientific grouping of forest types should remain s a research priority.

Another factor that may contribute to variations in FBC estimates results from differences in the length of age class intervals among and within forest types. Some slow-growing tree species have relatively lengthy time periods for delineating age class intervals. For example, the period for young *Pinus koraiensis* in natural forests is 60 years, while that for the young forest stage of natural *Pinus sylvestnis* var. *mongolica* and hardwood forests is 40 years. At the same time, some fast growing tree species have very short age class intervals, e.g., 10 years for young *Populus spp.* Selecting what age forests in which to establish sample plots to represent the age class to which they belong will clearly influence the calculation, and ultimately the accuracy, of parameters for FBC estimation. If the number of sample plots for slow- and fast-growing forests in a given age class is the same, the carbon estimate for the fast-growing forest may be more accurate. For example, 10 plots in young *Populus* (10-year class intervals) will likely yield a somewhat more accurate estimate (1 plot for every age increment) than the same number of plots in young *Pinus koraiensis* (60-year age class interval). Additionally, for the same tree species, the time periods for a given age class differs for natural forests and plantations. For example, the age class interval for young *Pinus koreansis* in plantations is 40 years, but in natural forests it is 60 years. Thus for *Pinus koreansis* of age 40–60 years, the age of young natural forests essentially equivalent to that of middle-age plantation forests. This implies that the time period for age classes should also be considered in determining the required number of sample field plots for different forest types.

## Conclusion

Estimates of forest biomass carbon storage in China have varied due to different methods used in the assessments. Young and middle-age forests account for a high proportion of forests in Liaoning Province, which in turn contributes to wide differences in estimates of forest biomass from different methods. In Liaoning Province, the highest estimates of forest biomass carbon (FBC) are generated by the mean biomass density method (MBM) and the lowest estimates by the mean ratio method (MRM); while the most realistic estimates are yielded via the continuous biomass expansion factor (CBM) method. Among all FBC estimates derived from different approaches and methods, significant differences originated with respect to young and middle-age forests. For Liaoning Province, with a large proportion of forest area in young and middle-age forests, the CBM method within the forest group with age class approach, which takes timber volume as a function of BEF and incorporates effects of forest age, forest density and forest site quality in Liaoning Province, designed in this study is more realistic and thus more appropriate for estimating forest biomass.

In general, forests in Liaoning Province are a carbon sink, while the largest FBC occurred in middle-age forests, the average carbon density decreased in these forests during the period of 1980–2010. The longer time periods in age classes for slow growing forest types enhanced the uncertainty of FBC estimates by CBM-forest type with age class, and future studies should pay more attention to the time periods for age classes in BEF establishment.

## Supporting Information

File S1 **Supporting tables. Table S1. Parameters for calculating forest live-biomass density (Mg ha^−1^) via the continuous biomass expansion factor method [CBM].** Biomass density is expressed as a function of forest growing stock (v, m^3^ ha^−1^), BEF = a+b/v, where b (Mg ha^−1^) and a (Mg m^−3^) are constants for a forest group. Data are from 1073 field plots. n is sample size, and r^2^ is the coefficient of determination. **Table S2. Parameters to calculate forest biomass density (Mg m^−3^) by mean ratio method [MRM].** Data are from 1073 field plots. **Table S3. Parameters to calculate forest biomass density by the mean biomass density method [MBM] (BD, Mg ha^−1^).** Data are from 1073 field plots in table 1.
(DOCX)
