# Supplementary material for: Estimates of Forest Biomass Carbon Storage in Liaoning Province of Northeast China: A Review and Assessment
Source: PLoS One. 2014 Feb 25;9(2):e89572. doi: 10.1371/journal.pone.0089572 (PMC3934887; doi:10.1371/journal.pone.0089572)
Supplement: File S1 — Supporting tables. Table S1. Parameters for calculating forest live-biomass density (Mg ha−1) via the continuous biomass expansion factor method [CBM]. Biomass density is expressed as a function of forest growing stock (v, m3 ha−1), BEF = a+b/v, where b (Mg ha−1) and a (Mg m−3) are constants for a forest group. Data are from 1073 field plots. n is sample size, and r2 is the coefficient of determination. Table S2. Parameters to calculate forest biomass density (Mg m−3) by mean ratio method [MRM]. Data are from 1073 field plots. Table S3. Parameters to calculate forest biomass density by the mean biomass density method [MBM] (BD, Mg ha−1). Data are from 1073 field plots in table 1. (DOCX) [file pone.0089572.s001.docx]

Table S1. Parameters for calculating forest live-biomass density (Mg ha^-1^) via the continuous biomass expansion factor method [CBM]. Biomass density is expressed as a function of forest growing stock (v, m^3^ ha^−1^), BEF=a+ b/v, where b (Mg ha^−1^) and a (Mg m^−3^) are constants for a forest group. Data are from 1073 field plots. n is sample size, and r^2^ is the coefficient of determination.

| Forest groups | Age class | a | b | r^2^ | n | a | b | r^2^ |
| --- | --- | --- | --- | --- | --- | --- | --- | --- |
| *Pinus koraiensis* | Young | 0.5157 | 12.108 | 0.93 | 29 | 0.5551 | 8.415 | 0.96 |
|  | Middle-age | 0.5407 | 6.6311 | 0.95 | 17 |  |  |  |
| *Larix olgensis* | Young | 0.5953 | 0.2736 | 0.97 | 38 | 0.5957 | 5.6349 | 0.90 |
|  | Middle-age | 0.5416 | 1.2931 | 0.97 | 23 |  |  |  |
|  | Mature | 0.6484 | -7.8047 | 0.94 | 28 |  |  |  |
| *Populus* spp. | Young | 0.9184 | -6.4064 | 0.89 | 70 | 0.8954 | -2.8678 | 0.97 |
|  | Middle-age | 0.9609 | -4.8252 | 0.95 | 26 |  |  |  |
|  | Near-mature | 0.907 | 2.6243 | 0.93 | 13 |  |  |  |
|  | Mature | 0.8066 | 5.4233 | 0.95 | 20 |  |  |  |
| *Pinus sylvestnis* var. *mongolica* | Young | 0.7878 | -1.2748 | 0.90 | 19 | 0.6698 | -0.8725 | 0.90 |
|  | Middle-age | 0.687 | -1.2404 | 0.85 | 32 |  |  |  |
| *Robinia pseudoacacia* | Young | 0.6297 | 0.4345 | 0.99 | 16 | 0.6240 | 0.053 | 0.98 |
|  | Middle-age | 0.6302 | -0.5623 | 0.98 | 19 |  |  |  |
| *Quecus spp.* | Young | 1.4245 | -1.8345 | 0.96 | 57 | 1.3022 | 3.7602 | 0.98 |
|  | Middle-age | 1.285 | 2.1278 | 0.97 | 28 |  |  |  |
|  | Mature | 1.1798 | 2.7533 | 0.88 | 24 |  |  |  |
| *Pinus tabuliformis* | Young | 0.9061 | 5.7594 | 0.98 | 177 | 0.8258 | 6.5065 | 0.98 |
|  | Middle-age | 0.8125 | 6.949 | 0.97 | 145 |  |  |  |
|  | Near-mature | 0.8307 | 2.8585 | 0.97 | 76 |  |  |  |
|  | Mature | 0.9255 | -18.479 | 0.99 | 57 |  |  |  |
| Broadleaved mixed forest | Young | 0.8409 | 4.0973 | 0.98 | 22 | 0.7851 | 10.128 | 0.93 |
|  | Middle-age | 0.7816 | 6.6375 | 0.97 | 18 |  |  |  |
|  | Mature | 0.7233 | 22.442 | 0.80 | 32 |  |  |  |
| Coniferous and broadleaved mixed forest | All age classes | 0.8355 | 0.4384 | 0.92 | 29 | 0.8355 | 0.4384 | 0.92 |
| Coniferous mixed forest | All age classes | 0.5577 | 3.3193 | 0.97 | 15 | 0.5577 | 3.3193 | 0.97 |
| Other hardwood trees | All age classes | 1.2973 | 2.2923 | 0.97 | 43 | 1.2973 | 2.2923 | 0.97 |

Mature forest includes over-mature forest

Table S2. Parameters to calculate forest biomass density (Mg m^-3^) by mean ratio method [MRM]. Data are from 1073 field plots.

| Forest groups | (mg/m^3)^ | | | | | | | $\overline{BEF_{i}}$ |  |
| --- | --- | --- | --- | --- | --- | --- | --- | --- | --- |
|  | Young | Middle- age | | Near-mature | | Mature^*^ | |  |  |
| *Pinus koraiensis* | 0.565 (0.038) | 0.645  (0.223) | | -- | | -- | | 0.584  (0.123) |  |
| *Larix olgensis* | 0.763  (0.164) | 0.551  (0.103) | | 0.608  (0.078) | | | | 0.629  (0.146) |  |
| *Populus* spp. | 0.765  (0.164) | 0.856  (0.237) | | 0.877  (0.109) | | 0.865  (0.127) | | 0.804  (0.156) |  |
| *Pinus sylvestnis* var. *mongolica* | 0.759  (0.161) | 0.812  (0.150) | | -- | | -- | | 0.806  (0.152) |  |
| *Robinia pseudoacacia^2^* | 0.650  (0.064) | 0.618  (0.049) | | -- | | -- | | 0.626  (0.052) |  |
| *Quecus spp.* | 1.398  (0.191) | 1.389  (0.122) | | 1.272  (0.090) | | | | 1.355  (0.158) |  |
| *Pinus tabuliformis* | 1.039  (0.310) | 0.911  (0.184) | | 0.858  (0.139) | | 0.843  (0.079) | | 0.903  (0.173) |  |
| Other hardwood trees^3^ | 1.390  (0.148) | 1.348  (0.387) | | 1.294  (0.073) | | | | 1.350  (0.294) |  |
| Coniferous and broadleaved mixed forest | 0.911  (0.322) | 0.896  (0.186) | | 0.821  (0.152) | | | | 0.840  (0.297) |  |
| Coniferous mixed forest | 0.580 (0.103) | | | | | | | |  |
| Broadleaved mixed forest | 0.842  (0.097) | 0.836  (0.089) | | 0.851  (0.074) | | | | 0.846  (0.094) |  |
| Total |  | |  | |  | |  | | |

^*^: Includes over-mature forest

---: No data available in the forest inventory.

Table S3. Parameters to calculate forest biomass density by the mean biomass density method [MBM] (BD, Mg ha^-1^) . Data are from 1073 field plots in table 1.

| Forest gourps | BD_i_ (sd) | BD_ij_ (sd) | | | | |
| --- | --- | --- | --- | --- | --- | --- |
|  |  | Young | Middle-age | Near mature | | Mature* |
| *Pinus koraiensis* | 99.65(58.29) | 60.56(29.79) | 148.85(55.92) |  | | |
| *Larix olgensis* | 78.88(54.59) | 50.72(30.08) | 78.91(33.03) | 117.00(61.34) | | |
| *Populus* spp. | 66.84(36.77) | 37.92(29.65) | 39.23(28.47) | 72.99(52.46) | | 94.11(17.13) |
| *Pinus sylvestnis* var. *mongolica* | 62.85(32.69) | 34.21(31.02) | 45.19(21.05) |  | | |
| *Robinia pseudoacacia* | 29.59(13.10) | 14.03(11.10) | 24.60(13.50) | --- | | |
| *Quecus mongolica* | 106.48(76.02) | 59.07(56.51) | 125.25(67.89) | 196.00(33.65) | | |
| *Pinus tabuliformis* | 76.33(33.01) | 38.04(29.10) | 49.16(45.32) | 71.43(49.89) | 117.23(81.82) | |
| Other hardwood trees | 106.48(76.02) | 57.29(56.51) | 125.52(51.32) | 169.60.00(83.14) | | |
| Other softwood trees | 100.30(64.27) | 66.21(57.11) | 104.17(48.64) | 149.97(34.48) | | |
| Coniferous and broadleaved mixed forest | 76.79(60.15) | 12.85(12.55) | 61.65(49.19) | 125.45(38.67) | | |
| Coniferous mixed forest | 87.95(47.230 | 87.95(47.23) | | | | |
| Broadleaved mixed forest | 100.30(64.27) | 66.21(57.11) | 104.17(48.64) | 149.97(34.48) | | |

^*^: Includes over-mature forest

---: No data available in the forest inventory.
